# A Biomimetic Approach for the Creation of Two-Dimensional Microscale Surface Patterns: Creation of Isolated Immunological Synapses

**DOI:** 10.1155/2009/821308

**Published:** 2009-06-17

**Authors:** Eric Stern, David J. Mooney, Tarek M. Fahmy

**Affiliations:** ^1^Department of Biomedical Engineering, School of Engineering and Applied Sciences, Yale University, New Haven, CT 06511, USA; ^2^Department of Bioengineering, School of Engineering and Applied Sciences, Harvard University, Cambridge, MA 02138, USA; ^3^Department of Chemical Engineering, School of Engineering and Applied Sciences, Yale University, New Haven, CT 06511, USA

## Abstract

Current efforts in surface functionalization have not produced a robust technique capable of creating specific two-dimensional microscale geometrical arrays composed of multiple proteins. Such a capability is desirable for engineering substrates in sensing and cell patterning applications where at least two different protein functionalities in a specific configuration are required. Here we introduce a new approach for the creation of arrays of microscale geometries. We demonstrate our approach with a biomimetic structure inspired by the immunological synapse, a cell-cell interfacial structure characterized by two concentric rings of proteins: an outer adhesion protein structure and an inner recognition ligand core. The power of the technique lies in its ability to pattern any protein in any defined geometry as well as to create arrays in parallel.

## 1. Introduction

Bioactive surfaces consisting of patterned proteins in specific defined geometries are important for screening applications [1], sensing [1–5], the study of cell-cell interactions [2, 6, 7], and the creation of cellular networks [2, 5, 8–10] or cellular aggregates for tissue engineering applications [11, 12]. A long sought after capability is the ability to fabricate patterned surfaces with cellular-like interfacial features (e.g., cell recognition, adhesion, and stimulatory ligands) to enhance cell-substrate interactions. Such biomimetic approaches require surface engineering with multiple chemical functionalities to facilitate attachment of a variety of proteins and to date, methodologies have been lacking in the robust production of arrays with multiple functionalities. Although surfaces capable of binding proteins at high densities are commercially available [13], these slides are designed for protein microarray screening and, in turn, require method development if specific patterns are required.

Current molecular patterning techniques such as poly(dimethylsiloxane) (PDMS) molding [9, 10, 14] are harsh in their application, potentially compromising surface functionality. Several groups have developed photo- and chemically-sensitive materials for protein patterning [5, 7, 15–17]; however, the techniques all require noncommercially available molecules and most have not yet been demonstrated to be capable of patterning multiple bioactive proteins [15–17]. Novel patterning approaches have also been developed, including microfluidic-directed patterning [18] and dip-pen lithography (DPL) [3]. Despite their promise in a number of applications, serial techniques such as DPL are not currently suited for the creation of large patterns and arrays and microfluidic patterning places strict geometric limitations on patterns.

Here we present a novel, photolithographic-based approach to create surfaces consisting of arrays of functional patterned proteins that uses only commercially available materials, as opposed to previously proposed methods [7, 15–17, 19–21]. We demonstrate our approach with a biomimetic structure inspired by the immunological synapse, an immunologically complex structure that occurs after lymphocytes encounter antigen-presenting cells [22]. The interfacial geometry of this structure is characterized by two concentric rings of proteins: an outer adhesion protein structure and an inner recognition ligand core.

The spatial reorganization of molecules on the surface of immune system cells such as T cells is a hallmark of early T-cell responses to foreign invasion [22]. Following interaction with antigen-presenting cells (APCs) this spatial reorganization takes a specific form, termed an immunological synapse (IS), which facilitates the encoding of information dictating the magnitude of the immune response. The hallmark of the mature IS is a “bulls-eye” pattern, which consists of regions termed supramolecular activation clusters (SMACs) [23–25]. The central SMAC (cSMAC) consists of interactions between T-cell receptors (TCRs) on the T-cell and peptide-loaded major histocompatibility complexes (pMHCs) on the APC. The peripheral SMAC or pSMAC contains adhesion and costimulatory ligands. Sensitivity is achieved because clustering promotes a high avidity interaction in the cSMAC [23–25]. Thus, creation of APC mimics on a surface would not only facilitate the study of T cell-APC signaling but may enable a recapitulation of sensitive sensing mechanisms employed by T cells in recognition of antigen [6, 7]. 

Recently, the application of a specially synthesized photoresist was demonstrated for the successful patterning of a surface with IS-like domains [7]. Despite the utility of the approach, this technique is not applicable for the formation of specific and precise patterned two-dimensional IS structures as observed in biological interactions. A cellular-repellant moiety such as poly(ethylene glycol) (PEG) cannot be bound at the periphery of these stuructures, thus cells adhere across the entire substrate rather than specifically at the ISs, compromising the utility of the approach for specific detection [26, 27].

We demonstrate here a method capable of patterning functional antibodies and PEG in any defined geometry using only commercially available products. We use this technique to create a patterned IS with one antibody conjugated in the central region and another in the peripheral region of the IS, surrounded by poly(ethylene glycol) (PEG) [26, 27]. Our method thus produces a surface with three chemical functionalities, created by two successive steps of traditional photolithographic patterning [28] followed by functionalization with chemical cross-linkers. An attractive feature of this method is that subsequent conjugations to these groups with specific molecules after photoresist removal was performed entirely in aqueous buffers, thus protein functionality is not compromised.

## 2. Materials and Methods

### 2.1. Materials

Amine-functionalized microscope slides were purchased from VWR (cat. no. 16001-008) and were cut to 1 in^2^ pieces using a diamond scribe. Photomasks were purchased from Benchmark Technologies and Photo Sciences, Inc and transparency photomasks were purchased from CAD Art Services. All photoprocessing steps were performed in a Class 1000 cleanroom (Harvard Center for Nanoscale Systems). All chemicals used for cleanroom processing were of cleanroom grade. The photoresist used for all processing was AZ 5214-E (MicroChem), an inverting resist. Glutaraldehyde, hydroxylamine, dimethyl sulfoxide (DMSO), phosphate buffered saline (PBS), ethylenedinitrilotetraacetic acid (EDTA), sodium cyanoborohydride, the fluorescein-5-isothiocyanate (FITC) conjugate of bovine serum albumin (BSA-FITC), and mouse IgA were purchased from Sigma-Alrich; *N*-succinimidyl-S-acetylthioacetate (SATP), *N*-(6-(biotinamido)hexyl)-3′-(2′-pyridyldithio)-propionamide (biotin-HPDP), sulfosuccinimidyl acetate (NHS-acetate), *N*-hydroxysulfosuccinimide (NHS), and 1-ethyl-3-(3-dimethylaminopropyl)carbodiimide (EDC) were purchased from Pierce Biotechnology; amine-terminated poly(ethylene glycol) (NH_2_-PEG; 10 kDa) and aldehyde-terminated PEG (CHO-PEG; 10 kDa) were purchased from Polysciences, Inc; FITC, AlexaFluor 568 hydrazide sodium salt (AlexaFluor 568), AlexaFluor 568 C_5_ maleimide (AlexaFluor 568 maleimide), streptavidin, and atto565-biotin were purchased from Invitrogen. Mouse IgG was obtained from Equitech-Bio. Antimouse-IgG-FITC was purchased from Zymed, Inc. All other antibodies were purchased from BD Biosciences.

### 2.2. Alignment Mark Deposition

Liftoff processing was used to pattern metal alignment marks on the slides for subsequent photolithographic steps. Amine-functionalized slides were cleaned with acetone and isopropanol (Baker Chemical Co.) and dried with nitrogen. Photoresist (AZ 5214-E) was spun on the slide at 3000 rpm using a Headway spinner. The slide was then baked at 90°C, exposed with the photomask using an AB-M maskaligner, baked at 110°C, and finally flood exposed. The slide was then developed in MF319 (MicroChem), washed with deionized water (DI; 18 MΩ), and blown dry with nitrogen. The slides were then loaded into a Sharon Systems Electron-Beam Evaporator, where a 100 nm titanium (99.9%, Kurt J. Lesker Co.) deposition was performed. After unloading, the slide was sonicated in acetone to remove the remaining photoresist, leaving only the metal alignment marks. Subsequent patterning steps followed the same procedure (without metal evaporation) with the exceptions that the photoresist was spun at 5000 rpm and that acetone was used to remove the photoresist without sonication.

### 2.3. Glutaraldehyde Conjugation

The slide containing the first layer of photoresist was treated for 1 hour at room temperature (RT) with a 0.01 M solution of glutaraldehyde in 0.1 M sodium acetate buffer, pH 5.5, with 1 mM sodium cyanoborohydride. Sodium cyanoborohydride was required for reduction of the unstable imine (C=N) bond. For all conjugation steps, the slides were placed face-up at the bottom of a 100 mL beaker with ~10 mL solution (enough to completely immerse the slide in the solution) and the beaker was covered with parafilm and placed on a shaker table. After each conjugation, the slide was removed, rinsed with DI, and blown dry with nitrogen (note after protein conjugation steps the slides were rinsed with PBS and not allowed to dry.

### 2.4. SATP Conjugation and Deprotection

*N*-Succinimidyl-*S*-acetylthiopropionate (SATP; 2 mg) was dissolved in 0.5 mL DMSO, which was added to 9.5 mL PBS and the slide containing the second layer of photoresist was treated for 30 minutes at RT with this solution. After washing, the slide was treated with a solution of 0.5 M hydroxylamine and 25 mM EDTA in PBS, pH 7.4, for 2 hours at RT to deprotect the sulfhydryl groups.

### 2.5. NH_2_-PEG Conjugation

Slides were treated with a 1% (w/v) solution of NH_2_-PEG in PBS, pH 7.4, with 1 mM sodium cyanoborohydrate for 4–6 hours at RT.

### 2.6. Biotin-HPDP Conjugation

*N*-(6-(Biotinamido)hexyl)-3′-(2′-pyridyldithio)-propionamide (Biotin-HPDP; 1 mg) was dissolved in 0.5 mL of DMSO, which was added to 9.5 mL of a 1 mM solution of EDTA in PBS, pH 7.4. The slide was treated with this solution for 1 hour at RT. We obtained more repeatable results when the biotin-HPDP conjugation followed that with NH_2_-PEG.

### 2.7. Fluorescent Antibody Conjugate Preparation

Mouse IgG, biotinylated-mouse IgA, antimouse IgG-FITC, and anti-LFA-1-FITC were used as received. Antimouse-IgA was dissolved to 1 mg/mL in PBS, pH 7.4, AlexaFluor 568 hydrazide was added to achieve a final concentration of 0.1 mg/mL, and 1-ethyl-3-(3-dimethylaminopropyl)carbodiimide hydrochloride (EDC) was added at 10 mg/mL. The reaction proceeded for 1 hour at RT with shaking and protected from light. A 50 mL Amicon Ultra (Millipore, cat. #UFC905024) centrifugal filter tube with a 50000 MW cutoff was used to remove unconjugated dye (4 30-minute spins at 3000 rpm at 4°C) and the resulting antimouse-IgA-AlexaFluor568 was diluted to 1 mg/mL. The same procedure was used for AlexaFluor 568 conjugation to biotinylated-anti-CD3*ε*. Conjugates were stored at 4°C.

### 2.8. NHS/EDC Coupling

Mouse IgG or anti-LFA-1-FITC was dissolved to 10 *μ*g/mL in PBS with 10 mg/mL EDC and 5 mg/mL N-hydroxysulfosuccinimide (sulfo-NHS) and reacted with the slide for 1 hour at RT. Washes with protein bound on the chip took place in PBS and the slides were not dried.

### 2.9. Streptavidin-Ac Preparation

Acetylated streptavidin was used because we found this protein to minimize nonspecific binding on the PEG surface (as compared with pure streptavidin, avidin, and neutravidin). To prepare the acetylated streptavidin, 5 mg/mL (sulfosuccinimidyl acetate) NHS-acetate was added to 1 mg/mL streptavidin in PBS, pH 7.4. The reaction proceeded for 1 hour at RT and the streptavidin conjugate was purified using a 50 mL Amicon Ultra centrifugal filter tube with a 10000 MW cutoff (4 30-minute spins at 3000 rpm at 4°C). The conjugate (streptavidin-Ac) was stored at 4°C.

### 2.10. Streptavidin-Ac Conjugation

The slides were treated with 10 *μ*g/mL streptavidin-Ac in PBS, pH 7.4, for 1 hour at RT. This conjugation does not affect biotin binding at the concentrations used in this work.

### 2.11. Biotinylated Antibody Conjugation

Biotinylated-mouse IgG or biotinylated anti-CD3*ε*-AlexaFluor568 were dissolved to 50 *μ*g/mL in PBS and reacted with the slide for 1 hour at RT (the latter was protected from light). When this reaction was completed, slides treated with anti-LFA-1-FITC and biotinylated-anti-CD3*ε*-AlexaFluor 568 were dried (gently) and viewed under a fluorescent microscope (Olympus IX81, equipped with the Carv-II unit from BD Biosciences). The low-intensity areas in the bottom of some images (notably Figure 3(d)) are due to the directional nature of the stream of air during this drying step.

### 2.12. Immunoactivity Study

Slides containing mouse IgG and biotinylated-mouse IgA were treated with a PBS, pH 7.4, solution containing 50 *μ*g/mL antimouse-IgG FITC and 50 *μ*g/mL antimouse-IgA-AlexaFluor 568 for 1 hour at RT. These slides were subsequently dried (gently) and viewed under a fluorescent microscope.

## 3. Preparation of Isolated ISs

### 3.1. Surface Chemistry Demonstrations

**Figure 1:**
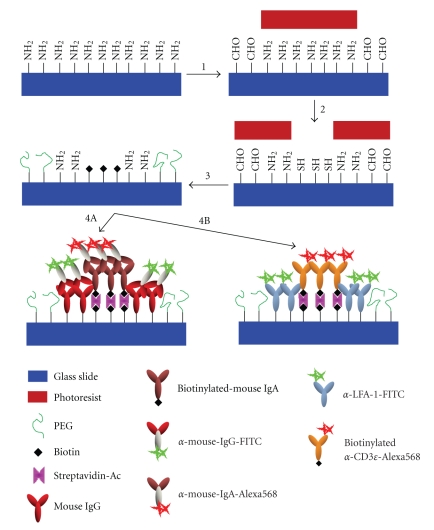
Sequence of photopatterning and chemical treatment steps for binding antibodies and PEG to create surface APC mimics. The steps are thoroughly discussed in the text. Briefly, Step  1 shows the first layer of photoresist patterning followed by subsequent glutaraldehyde conjugation to exposed amine (NH_2_) groups. Step  2 is the second photopatterning (following removal of the first layer) with subsequent thiol (SH) conjugation to exposed NH_2_ groups. In Step  3, PEG is bound to aldehyde (CHO) groups and free SH groups are biotinylated. Step  4A demonstrates IgG conjugation to exposed NH_2_ groups and biotinylated-IgA binding to surface-bound streptavidin-Ac. Antibody recognition of specific antigens enables fluorescently conjugated antibodies to specifically bind active IgG and IgA. Step  4B shows a similar binding of two fluorescently-labeled IgGs, which create an artificial IS in this geometry.

**Figure 2:**
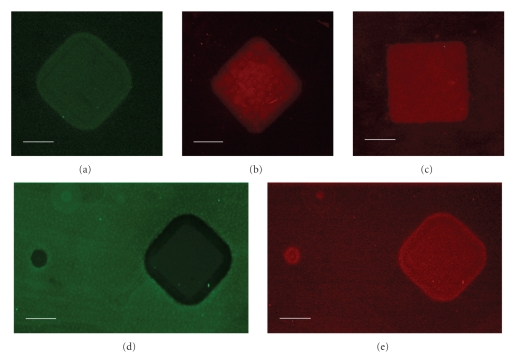
Fluorescent micrographs demonstrating conjugation of coupling molecules to patterned surfaces. Panels are described in detail in the text; briefly functionalization with (a) aldehyde groups, (b) sulfhydryl groups, (c) biotin molecules, and ((d) and (e)) proteins are illustrated. The patterned squares in all micrographs are 25 × 25 *μ*m^2^ and the white scale bars represent 10 *μ*m.

In Figure 1 (Step  1), photoresist spun on amine-functionalized microscope slides was patterned and the exposed amine groups were converted to aldehydes with glutaraldehyde. Subsequent removal of the photoresist left a slide with two functionalities, with the aldehydes defining areas between the ISs. Successful glutaraldehyde conjugation was first demonstrated using a transparency photomask consisting of an array of 25 × 25 *μ*m^2^ squares (clear on a dark background), followed by gluaraldehyde treatment. Aldehyde functionality was thereby conferred to all areas of the chip but these squares. The photoresist was subsequently removed and the slide was treated with aldehyde-reactive PEG (NH_2_-PEG). After subsequent treatment with fluorescein-5-isothiocyanate (FITC), a fluorescent amine-reactive probe, the slides were fluorescently imaged under a GFP filter (Figure 2(a)). The square fluoresces due to the conjugation of FITC to the amine surface but the formation of an amide bond between the surface aldehyde and free amine of the NH_2_-PEG prevents FITC binding in areas surrounding the square.

In Step  2, a second photoresist layer was applied and patterned and the available amine groups were reacted with SATP, resulting in the conversion of exposed amine groups to protected thiols [29]. Photoresist removal with acetone followed by thiol deacetylation with hydroxylamine thus resulted in a slide with three chemical functionalities, where the thiols defined the cSMAC and the remaining amines defined the pSMAC. In order to demonstrate successful SATP conjugation, slides were photopatterned with a transparency mask consisting of an array of 25 × 25 *μ*m^2^ squares (dark on a clear background) followed by SATP conjugation and subsequent deprotection. This resulted in the presence of sulfhydryl groups only in the squares. The photoresist was then removed and slides were treated with amine-reactive PEG (CHO-PEG). A sulfhydryl-reactive fluorescent probe (AlexaFluor 568 maleimide) was then conjugated to the slides and subsequent fluorescent imaging using a TRITC filter showed that the high fluorescence intensity was confined to the squares, demonstrating that sulfhydryl groups are present in these areas (Figure 2(b)).

As shown in Step  3, in order to eliminate nonspecific adsorption of cells to nonsynaptic regions of the slide, NH_2_-PEG (10 kDa) was conjugated to the exposed aldehydes. Next, biotin-HPDP was conjugated to the thiols in the cSMAC region, thereby biontinylating this area and readying the slide for protein conjugation [29]. Biotin-HPDP conjugation was demonstrated by processing slides with a transparency mask containing an array of 25 × 25 *μ*m^2^ squares (dark on a clear background), followed by SATP conjugation and deprotection. After photoresist removal slides were treated with CHO-PEG, followed by biotin-HPDP. The slides were then treated with acetylated streptavidin (streptavidin-Ac), followed by treatment with atto565-biotin, a fluorescently conjugated biotin molecule. Slides were fluorescently imaged under a TRITC filter (Figure 2(c)). As in Figure 2(b), the high fluorescence intensity is confined to the square, demonstrating the successful conjugation of biotin-HPDP to the sulfhydryl groups, as well as subsequent streptavidin-Ac and atto565-biotin binding.

### 3.2. Protein and Antibody Conjuation

Protein conjugation to slides using EDC/sulfo-NHS coupling [29] (Step  4A) was demonstrated by processing slides with a transparency mask containing an array of 25 × 25 *μ*m^2^ squares (dark on a clear background), followed by SATP conjugation and deprotection. After photoresist removal, slides were treated with biotin-HPDP, as described above. BSA-FITC was then added to the chip with 10 mg/mL EDC and 5 mg/mL sulfo-NHS. The slides were then treated first with streptavidin-Ac and next with atto565-biotin. Fluorescence imaging was performed with both GFP (Figure 2(d)) and TRITC (Figure 2(e)) filters (images are from the same array point). The high fluorescence intensity in the region surrounding the square in Figure 2(d) indicates that BSA-FITC binding was confined to this region, while the high fluorescence intensity in the square in Figure 2(e) shows that atto565-biotin binding was confined to this region.

**Figure 3:**
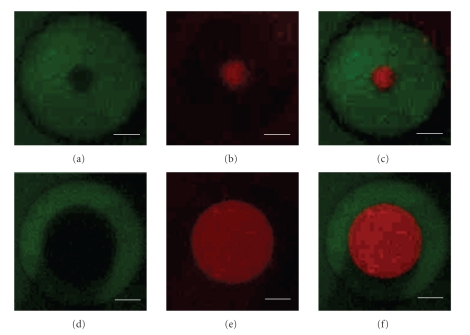
Fluorescent micrographs showing bound antimouse IgG-FITC ((a), (d); GFP filter), bound antimouse IgA-AlexaFluor568 ((b), (e); TRITC filter), and an overlay of both channels ((c), (f)). The white scale bars represent 10 *μ*m. The images are from two representative patterns of different dimensions fabricated in parallel on the same chip. Experiments were repeated three times with similar results.

We next demonstrated the ability of this approach to pattern the surface with functional antibodies (Step  4A). We first conjugated mouse-immunoglobulin G (IgG) [30] to the exposed amine groups through standard EDC/sulfo-NHS chemistry. The slide was then treated with acetylated streptavidin and subsequently with biotinylated mouse-immunoglobulin A (IgA). Unlike other patterning techniques [15–17], our approach does not require the presence of blocking agents during the final conjugation steps. The slide was next incubated with an FITC conjugate of antimouse-IgG and an Alexa Fluor 568 conjugate of antimouse-IgA, washed, and imaged. The fluorescent micrographs in Figure 3 demonstrate the functionality of the conjugated antibodies as well as their segregation into different regions dictated by the surface functional groups. This also enables the creation of arrays of patterns with varying absolute and relative dimensions.

**Figure 4:**
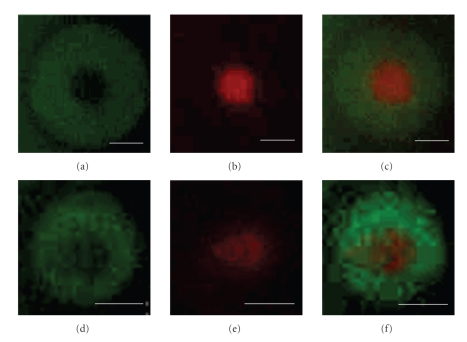
Fluorescent micrographs showing bound anti-LFA-1-FITC ((a), (d); GFP filter), bound anti-CD3*ε*-AlexaFluor568 ((b), (e); TRITC filter), and an overlay of both channels ((c), (f)). The white scale bars represent 5 *μ*m. The images are from two representative patterns of different dimensions fabricated in parallel on the same chip. Experiments were repeated three times with similar results.

This chemistry can be utilized to functionalize slides with physiologically relevant antibodies to mimic the IS. For this purpose we chose antibodies to two ligands known to be sequestered in the IS: an antibody to leukocyte function-associated antigen 1 (anti-LFA-1) as the adhesion promoter for the pSMAC and an antibody to the *ε* subunit of cluster of differentiation 3 (anti-CD3*ε*), a well-established T-cell stimulus, for the cSMAC [7, 25]. As shown in Step  4B of Figure 1, antimouse-LFA-1 (FITC conjugate) was coupled to the free amines in the pSMAC region, followed by treatment of the slide with biotinylated antimouse-CD3*ε* (AlexaFluor 568 conjugate), which bound the streptavidin in the cSMAC (Figure 4). We chose to use streptavidin as the linker chemistry in the cSMAC because biotinylated T-cell stimuli (pMHCs and antibodies) are readily available. The dimensions of the SMAC mimics shown in Figure 4 are similar to those of T cells [23–25]. The protein patterns were stable for >2 months when slides were stored in PBS at 4°C.

## 4. Conclusions

The power of this technique is its simplicity of implementation and ability to create massive arrays in parallel of specifically-defined two-dimensional protein patterns without the need for blocking agents during conjugation steps, enabling cell patterning and the study of cellular interactions. Although we focused on the formation of surface SMAC mimics, any geometry is easily realized—much like integrated circuits—by designing and using a specific photomask set. Additionally, by combining this approach with that of microarray printing [13], arrays can be created in which each element contains a different functionality, enabling high-throughput screening.

*Supporting Information Available*. An alternative route to surface IS functionalization is given in addition to two figures, one a schematic describing the method and a second showing fluorescent optical micrographs of representative ISs patterned using the approach.

## Supplementary Material

An alternative route to surface IS functionalization is given in addition to two figures; one is a schematic diagram describing the method and the second is showing fluorescent optical micrographs of representative ISs patterned using the approach.

## References

[1] Houseman BT, Huh JH, Kron SJ, Mrksich M (2002). Peptide chips for the quantitative evaluation of protein kinase activity. *Nature Biotechnology*.

[2] Folch A, Toner M (2000). Microengineering of cellular interactions. *Annual Review of Biomedical Engineering*.

[3] Salaita K, Wang Y, Mirkin CA (2007). Applications of dip-pen nanolithography. *Nature Nanotechnology*.

[4] John PMSt, Davis R, Cady N, Czajka J, Batt CA, Craighead HG (1998). Diffraction-based cell detection using a microcontact printed antibody grating. *Analytical Chemistry*.

[5] Feng CL, Embrechts A, Bredebusch I (2007). Tailored interfaces for biosensors and cell-surface interaction studies via activation and derivatization of polystyrene-*block*-poly(*tert*-butyl acrylate) thin films. *European Polymer Journal*.

[6] Mossman KD, Campi G, Groves JT, Dustin ML (2005). Immunology: altered TCR signaling from geometrically repatterned immunological synapses. *Science*.

[7] Doh J, Irvine DJ (2006). Immunological synapse arrays: patterned protein surfaces that modulate immunological synapse structure formation in T cells. *Proceedings of the National Academy of Sciences of the United States of America*.

[8] Welle A, Horn S, Schimmelpfeng J, Kalka D (2005). Photo-chemically patterned polymer surfaces for controlled PC-12 adhesion and neurite guidance. *Journal of Neuroscience Methods*.

[9] Oliva AA, James CD, Kingman CE, Craighead HG, Banker GA (2003). Patterning axonal guidance molecules using a novel strategy for microcontact printing. *Neurochemical Research*.

[10] Leng T, Wu P, Mehenti NZ (2004). Directed retinal nerve cell growth for use in a retinal prosthesis interface. *Investigative Ophthalmology and Visual Science*.

[11] Sands RW, Mooney DJ (2007). Polymers to direct cell fate by controlling the microenvironment. *Current Opinion in Biotechnology*.

[12] Cao L, Mooney DJ (2007). Spatiotemporal control over growth factor signaling for therapeutic neovascularization. *Advanced Drug Delivery Reviews*.

[13] Zhu H, Snyder M (2003). Protein chip technology. *Current Opinion in Chemical Biology*.

[14] Patel N, Bhandari R, Shakesheff KM (2000). Printing patterns of biospecifically-adsorbed protein. *Journal of Biomaterials Science, Polymer Edition*.

[15] Husemann M, Morrison M, Benoit D (2000). Manipulation of surface properties by patterning of covalently bound polymer brushes. *Journal of the American Chemical Society*.

[16] Lee K, Pan F, Carroll GT, Turro NJ, Koberstein JT (2004). Photolithographic technique for direct photochemical modification and chemical micropatterning of surfaces. *Langmuir*.

[17] Pan F, Wang P, Lee K, Wu A, Turro NJ, Koberstein JT (2005). Photochemical modification and patterning of polymer surfaces by surface adsorption of photoactive block copolymers. *Langmuir*.

[18] Delamarche E, Juncker D, Schmid H (2005). Microfluidics for processing surfaces and miniaturizing biological assays. *Advanced Materials*.

[19] Juncker D, Schmid H, Delamarche E (2005). Multipurpose microfluidic probe. *Nature Materials*.

[20] Lee YS, Mrksich M (2002). Protein chips: from concept to practice. *Trends in Biotechnology*.

[21] Stern E, Jay S, Bertram J (2006). Electropolymerization on microelectrodes: functionalization technique for selective protein and DNA conjugation. *Analytical Chemistry*.

[22] Charles J, Janeway A, Travers P, Walport M, Shlomchik MJ (2005). *Immunobiology: The Immune System in Health and Disease*.

[23] Monks CRF, Freiberg BA, Kupfer H, Sciaky N, Kupfer A (1998). Three-dimensional segregation of supramolecular activation clusters in T cells. *Nature*.

[24] Grakoui A, Bromley SK, Sumen C (1999). The immunological synapse: a molecular machine controlling T cell activation. *Science*.

[25] Huppa JB, Davis MM (2003). T-cell-antigen recognition and the immunological synapse. *Nature Reviews Immunology*.

[26] Sharma S, Johnson RW, Desai TA (2004). Evaluation of the stability of nonfouling ultrathin poly (ethylene glycol) films for silicon-based microdevices. *Langmuir*.

[27] Leckband D, Sheth S, Halperin A (1999). Grafted poly(ethylene oxide) brushes as nonfouling surface coatings. *Journal of Biomaterials Science, Polymer Edition*.

[28] Madou MJ (2009). *Fundamentals of Microfabrication and Nanotechnology*.

[29] Hermanson GT (1996). *Bioconjugate Techniques*.

[30] Voet D, Voet JG (1995). *Biochemsitry*.

