# Supplementary material for: A Biomimetic Approach for the Creation of Two-Dimensional Microscale Surface Patterns: Creation of Isolated Immunological Synapses
Source: Int J Biomater. 2009 Jun 17;2009:821308. doi: 10.1155/2009/821308 (PMC2814121; doi:10.1155/2009/821308)

Supporting Information for: A Biomimetic Approach for the Creation of Two-Dimensional Microscale Surface Patterns: Creation of Isolated Immunological Synapses

Eric Stern, David J. Mooney, Tarek M. Fahmy

Alternative Route. Figure S1 shows a similar, alternative route: during Step 2, rather than converting the exposed amines to sulfhydryls, they are directly biotinylated. Although we were unsuccessful using all available amine-reactive biotin conjugates [sulfosuccinimidyl-6-(biotinamido) hexanoate (NHS-LC-biotin), Pierce Biotechnology], by first using glutaraldehyde to convert the amine groups to aldehydes and subsequently (prior to photoresist removal) using biotin-LC-hydrazide, we were able to successfully biotinylate the cSMAC region, as shown in the fluorescent micrographs in Figure S2 (the white scale bars represent 10 μm). All reactions were performed as detailed in the *Materials and Methods* section. Experiments were repeated three times with similar results.

Figure S1

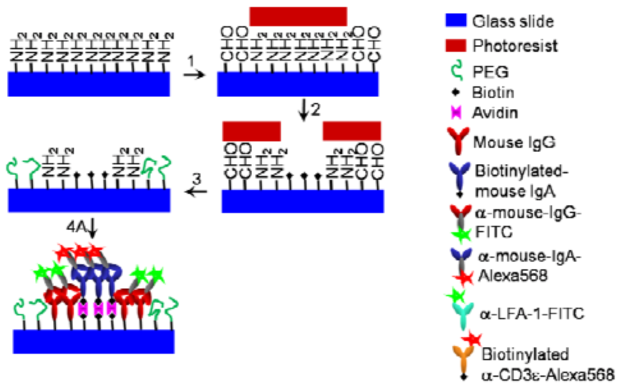

Figure S2

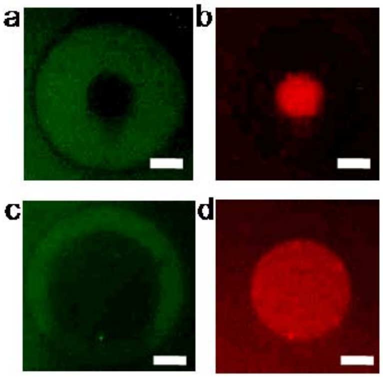

Supplement: Supplementary file 1 — An alternative route to surface IS functionalization is given in addition to two figures; one is a schematic diagram describing the method and the second is showing fluorescent optical micrographs of representative ISs patterned using the approach. [file 821308.f1.pdf]
